# Favorable Response to CD34+ Cell Therapy Is Associated with a Decrease of Galectin-3 Levels in Patients with Chronic Heart Failure

**DOI:** 10.1155/2019/8636930

**Published:** 2019-12-09

**Authors:** Gregor Poglajen, Jus Ksela, Sabina Frljak, Gregor Zemljic, Elizabeta Boznar Alic, Andraz Cerar, Bojan Vrtovec

**Affiliations:** ^1^Advanced Heart Failure and Transplantation Center, University Medical Center Ljubljana, Slovenia; ^2^Faculty of Medicine, Ljubljana, Slovenia; ^3^Department of Cardiovascular Surgery, University Medical Center Ljubljana, Slovenia; ^4^Department of Clinical Biochemistry, University Medical Center Ljubljana, Slovenia

## Abstract

**Background:**

Galectin-3 plasma levels (gal-3) were shown to correlate with the scar burden in chronic heart failure (CHF) setting. As scar burden predicts response to stem cell therapy, we sought to explore a correlation between gal-3 and response to CD34+ cell transplantation in patients with CHF.

**Methods:**

We performed a post hoc analysis of patients, enrolled in 2 prospective trials investigating the clinical effects of CD34+ cell therapy in patients with ischemic cardiomyopathy (ICMP) and nonischemic dilated cardiomyopathy (DCMP). CD34+ cells were mobilized by G-CSF, collected via apheresis, and injected transendocardially using NOGA system. Patients were followed for 3 months and demographic, echocardiographic, and biochemical parameters and gal-3 were analyzed at baseline and at follow-up. Response to cell therapy was defined as an LVEF increase of ≥5%.

**Results:**

61 patients were included in the analysis. The mean age of patients was 52 years and 83% were male. DCMP and ICMP were present in 69% and 31% of patients, respectively. The average serum creatinine was 86 ± 23 *μ*mol/L, NT-proBNP 1132 (IQR 350-2279) pg/mL, and LVEF 30 ± 6%. Gal-3 at baseline and at 3 months did not differ significantly (13.4 ± 5.5 ng/mL vs. 13.1 ± 5.8 ng/mL; *p* = 0.72), and there were no differences in baseline gal-3 with respect to heart failure etiology (15.1 ± 7.2 ng/mL in ICMP vs. 12.7 ± 4.3 ng/mL in DCMP; *p* = 0.12). Comparing responders (*N* = 49) to nonresponders (*N* = 18), we found no differences in baseline gal-3 (13.6 ± 5.7 ng/mL vs. 13.2 ± 4.9 ng/mL; *p* = 0.80). However, responders had significantly lower gal-3 at 3-month follow-up (12.1 ± 4.0 ng/mL vs. 15.7 ± 8.4 ng/mL; *p* < 0.05). Also, responders demonstrated a significant decrease in gal-3 over 3 months, while in nonresponders, an increase in gal-3 occurred (−1.5 ± 5.4 ng/mL vs. +2.7 ± 4.3 ng/mL; *p* = 0.01).

**Conclusions:**

In patients with chronic heart failure undergoing CD34+ cell therapy, a decrease in galectin-3 plasma levels is associated with beneficial response to this treatment modality. Further prospective data is warranted to confirm our findings and to deepen our understanding of the role of gal-3 in the field of stem cell therapy.

## 1. Introduction

In patients with chronic heart failure, stem cell therapy with the use of different stem cell types and different routes of stem cell delivery has recently been shown to improve myocardial performance, decrease neurohumoral activation, and improve exercise capacity and quality of life, and there was even a signal towards improved survival in this patient cohort [1–9].

Although these results are encouraging currently, there is still no consensus regarding the optimal patient selection for this treatment strategy. Published data suggest a variable response to stem cell therapy with, depending on the definition of response, 30-60% of patients failing to adequately respond to this treatment option [2, 3, 9, 10]. These differences may be related to several patient- and procedure-related factors, such as the underlying cause and the duration of heart failure, heart failure stage and end-organ function, type and quantity of injected stem cells, and the method of stem cell delivery (intracoronary vs. transendocardial vs. transepicardial) amongst others [3, 9, 11].

Additionally, using electroanatomical data, it was demonstrated that scar burden of the failing myocardium may also play a significant role in determining the response to stem cell therapy [10]. However, invasive scar burden assessment is not feasible for routine clinical use and accurate noninvasive determination of myocardial scar burden (with myocardium imaging or serum biomarkers) that could be used to improve patient selection for stem cell therapy remains a challenge.

Recently, our group explored the potential of serum biomarkers to predict response to CD34+ cell therapy in patients with nonischemic dilated cardiomyopathy [12]. Our data failed to establish a correlation between the response to stem cell therapy, defined as improvement of LVEF ≥ 5% at 3 months post stem cell injection, and factors, traditionally related to poor prognosis in heart failure population, such as patient age, gender, exercise capacity, baseline left ventricular ejection fraction (LVEF), and baseline serum levels of NT-proBNP. However, we were able to show that responders on average mobilized higher number of CD34+ cells (119 ± 68 × 106 vs. 74 ± 55 × 106; *p* = 0.03) and had higher myocardial cell retention rates (14 ± 5% vs. 9 ± 5%; *p* = 0.01). Additionally, we were able to identify several immunologic and nonimmunologic biomarkers and metabolic factors, associated with myocardial inflammation, stem cell mobilization and retention, cell growth, and metabolic alterations in the failing myocardium that were positively or negatively independently associated with the response to CD34+ cell therapy [12]. Importantly, biomarkers that may be directly related to myocardial fibrosis were not addressed in this analysis.

In the past decade, galectin-3 has been widely explored in chronic heart failure setting and has been established as a biomarker independently associated with left ventricular remodeling and adverse prognosis in terms of heart failure-related hospitalizations and increased mortality in this patient cohort [13–15]. Galectin-3 is a *β*-galactoside-binding lectin that is best known for its involvement in tumor growth and metastasis. In chronic heart failure, galectin-3 has been demonstrated to be involved in several pathophysiological processes, including myocardial inflammation and fibrosis, which are both pivotal mechanisms of heart failure development and progression [16].

Currently, the potential underlying mechanisms of the favorable response to stem cell therapy remain undefined. We therefore sought to explore a correlation between changes in galectin-3 plasma levels and response to transendocardial CD34+ cell transplantation in patients with chronic heart failure.

## 2. Methods

### 2.1. Patient Population

We performed a post hoc analysis of patients, enrolled in 2 prospective open-label trials investigating the clinical effects of transendocardial CD34+ cell therapy in patients with ischemic cardiomyopathy (ICMP, NCT01350310) and nonischemic dilated cardiomyopathy (DCMP, NCT02445534). The study protocol was approved by the National Medical Ethics Committee. Firstly, 125 patients who underwent stem cell therapy between January 1, 2013, and December 31, 2016, were screened for the study. Inclusion criteria were as follows: age (18-65 years), optimal medical management for ≥6 months, LVEF < 40%, and diagnosis of DCMP according to the European Society of Cardiology position statement [17] or diagnosis of ICM without any option for further percutaneous or surgical myocardial revascularization [18]. Patients with acute coronary syndrome or hospitalization for worsening heart failure requiring inotropic support within 12 months before stem cell therapy, patients with heart failure with preserved ejection fraction, patients who received repetitive stem cell application, and patients with end-stage renal disease or liver cirrhosis were excluded from the study. Chronic kidney disease (CKD) was defined as serum creatinine > 100 mmol/L. Ultimately, 61 patients met the proposed criteria and were included in the study and an informed consent was obtained from all patients before participation in the study.

### 2.2. Study Design

All patients underwent stem cell mobilization and collection as per institution's protocol as outlined in detail previously by our research group [2, 3, 19]. After 5-day granulocyte colony-stimulating factor therapy (5 *μ*g/kg BID), CD34+ cells were collected through apheresis of peripheral blood. Then, all patients underwent electroanatomical mapping and transendocardial CD34+ cell implantation. In patients who met the inclusion criteria, demographic, echocardiographic, and biochemical parameters and galectin-3 plasma levels were analyzed at the time of stem cell transplantation (baseline) and at 3-month follow-up.

### 2.3. Peripheral Blood Stem Cell Mobilization and Collection

All patients underwent stem cell mobilization and collection as described previously in detail [2, 3, 19, 20]. In short, following the methods of Vrtovec et al. [2, 3] and Poglajen et al. [19], bone marrow cells were mobilized into peripheral blood by daily subcutaneous injections of G-CSF (5 *μ*g/kg BID). On the fifth day, bone marrow mononuclear cells were collected via cytapheresis with Miltenyi cell separator (Miltenyi Biotec, Germany). Then immunoselection of collected cells was performed with Isolex 300i (Nexell Therapeutics Inc., CA). Selected CD34+ cells were stored in the cell collection bag and additionally concentrated to a final volume of 6 mL.

### 2.4. Electroanatomical Mapping and Transendocardial 34+ Cell Delivery

Electroanatomical mapping is a procedure performed with the Biosense NOGA system (Biosense Webster, Diamond Bar, CA). This platform allows for point-by-point analysis of left ventricular viability and local contractility [21]. Using this technique, maps of color-coded myocardial viability (unipolar voltage, UV) and regional myocardial contraction (linear shortening, LLS) and their corresponding bull's-eye maps, consisting of ≥200 sampling points, were generated for each patient prior to stem cell transplantation. Hibernating myocardium was defined as areas with UV ≥ 8.3 mV and LLS<6%; myocardial scar was defined as segments with UV < 8.3 mV and LLS < 6% [2, 3, 22]. Transendocardial delivery of cell suspension was done using the MyoStar (Biosense Webster) injection catheter. Each patient received 20 injections of stem cell suspension, 0.3 mL of cell suspension per injection; all injections were performed within the areas of myocardial hibernation (UV ≥ 8.3 mV and LLS < 6%).

### 2.5. Echocardiography, 6-Minute Walk Test, and NT-proBNP Measurements

The echocardiography data were recorded and analyzed at the end of the study by an independent echocardiographer who was blinded to patient's treatment status and the timing of the recordings. Left ventricular end-systolic volume and end-diastolic volume and LVEF were estimated using Simpson's biplane method, and left ventricular end-systolic dimension and end-diastolic dimension were measured in the parasternal long-axis view. All echocardiographic measurements were averaged for 5 cycles [2, 3]. In all patients, a 6-minute walk test was performed by a blinded observer according to the standard protocol [23]. All NT-proBNP assays were performed at a central independent laboratory, blinded to patient's clinical data using a commercially available kit (Roche Diagnostics, Mannheim, Germany).

### 2.6. Galectin-3 Measurement

Galectin-3 plasma levels were determined in an independent laboratory blinded to patient clinical data on stored specimens (drawn prior to G-CSF stimulation, stored at -80°C, thawed once) using an enzyme-linked immunosorbent assay (BG Medicine, Waltham, MA, USA). This assay quantitatively measures the concentration of human galectin-3 levels in EDTA plasma and has been shown to have high sensitivity (with a lower detection limit of 1.13 ng/mL) and no cross-reactivity with other members of the galectin family, or with other collagens, and no known interference by commonly used heart failure medications.

### 2.7. Definition of Clinical Response

Response to stem cell therapy was defined as an absolute increase in LVEF of ≥5% at 3 months after CD34+ cell therapy [2, 3]. The threshold of 5% was chosen based on meta-analysis of heart failure trials showing that a 5% increase in the mean EF change corresponded to a relative odds ratio of 0.86 (95% confidence interval (CI) 0.77-0.96) for mortality [24].

### 2.8. Statistical Methods and Analysis

Continuous variables are presented as mean (±SD) or median (interquartile range) where appropriate and categorical data are given as count (percent). Categorical variables were compared with the chi-squared test (or Fischer exact nonparametric test). All continuous variables were compared using Student's *t*-test and ANOVA (or Mann-Whitney nonparametric test). Statistical significance was assumed for *p* values of <0.05. All statistical analyses were performed with SPSS software (version 20.0).

## 3. Results

### 3.1. Patient Characteristics

**Figure 1:**
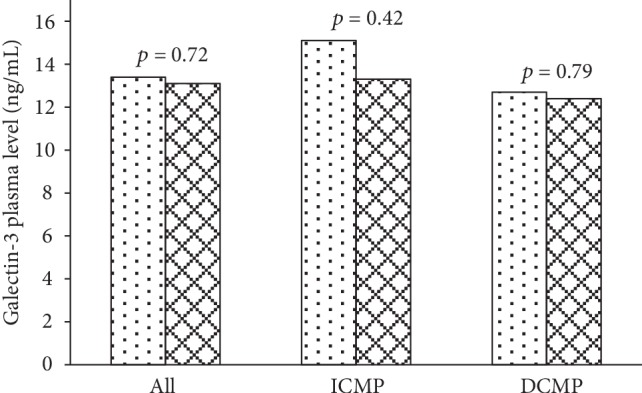
Comparison of plasma galectin-3 baseline (spotted bars) and follow-up (checkered bars) values found no differences when compared in the entire patient cohort and separately in patients with ICMP and DCMP.

Detailed baseline patient characteristics are outlined in Table 1. The majority of included subjects were middle-aged male patients with LVEF around 30%. Neurohumoral activation was significantly elevated with the median NT-proBNP serum levels reaching 1132 pg/mL. End-organ function was not significantly affected in any of patients and no significant electrolyte disturbances or complete blood count deviations were noted. All patients received optimal evidence-based heart failure therapy that was not altered during the study period. Looking at the entire patient cohort, galectin-3 plasma levels did not change significantly between baseline and 3-month follow-up (Figure 1).

### 3.2. Galectin-3 and Heart Failure Etiology

We further compared patients based on heart failure etiology. No changes were found between patients with ICMP (*N* = 19) and DCMP (*N* = 42) considering age, gender, LVEF, functional capacity, neurohumoral activation, or end-organ function. Additionally, there were no significant differences regarding heart failure medical management observed between the two groups with the exception of antiaggregation therapy which was significantly higher in the ICMP group as expected. Importantly, the two groups did not differ with respect to the galectin-3 plasma levels at the baseline and at 3-month follow-up (Figure 1).

### 3.3. Changes in Galectin-3 Levels and Response to CD34+ Cell Therapy

**Figure 2:**
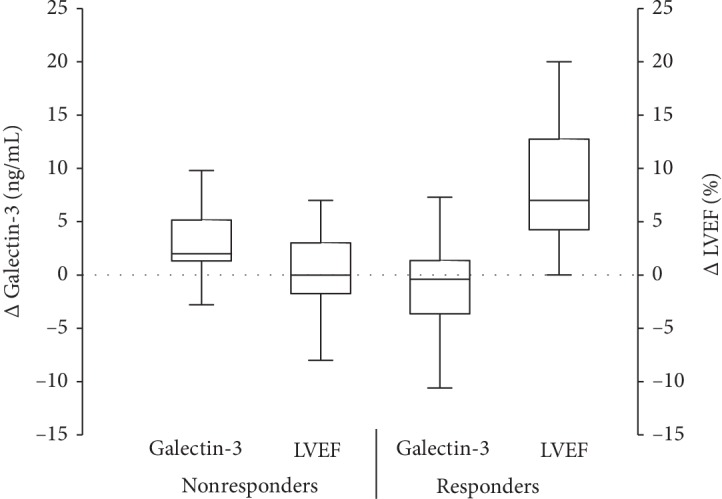
Comparison of plasma galectin-3 level changes between responders and demonstrated that galectin-3 serum levels decreased in responders but increased in nonresponders at 3-month follow-up. Furthermore, LVEF did not change significantly in nonresponders but increased significantly in responders.

We further stratified patients conditional to the response to CD34+ cell therapy (Table 2). The nonresponders (*N* = 18) and responders (*N* = 43) were similar with respect to age, gender, heart failure etiology, LVEF, and exercise capacity. Also, liver function tests, complete blood count, and heart failure medical therapy did not differ between the two groups. However, in comparison to responders, nonresponders had significantly higher serum levels of NT-proBNP and creatinine, higher RDW, and lower baseline LVEF. Considering galectin-3, baseline plasma levels did not differ between the two groups (Table 2). Importantly, galectin-3 plasma levels at 3 months were significantly lower in responders than in nonresponders. Furthermore, a net decrease in plasma galectin-3 levels was significantly higher in responders than in nonresponders in which plasma galectin-3 values actually increased (change in galectin-3: −1.5 ± 5.4 ng/mL in responders vs. +2.7 ± 4.3 ng/mL in nonresponders; *p* = 0.01) (Figure 2).

Univariate correlates of response to CD34+ cell therapy were subsequently analyzed in a multivariate model which showed that a decrease in galectin-3 plasma levels between baseline and 3-month follow-up and baseline serum NT‐proBNP > 1000 pg/mL were independent correlates of response to CD34+ cell therapy (Table 3).

## 4. Discussion

This is the first study evaluating a correlation between changes of galectin-3 plasma levels and a response to CD34+ cell therapy. Despite the lack of association between baseline galectin-3 plasma levels and a response to CD34+ cell therapy, we were able to demonstrate that within 3 months after the procedure galectin-3 plasma decreases significantly more in clinical responders to CD34+ cell therapy.

Galectin-3, a *β*-galactoside-binding lectin, is a protein that has recently been heavily implicated in the pathophysiology of myocardial fibrosis. While it can be secreted by injured cardiomyocytes, activated M2 macrophages are believed to represent the main source of galectin-3 in the failing myocardium. Galectin-3 has been shown to promote proliferation of fibroblasts and collagen I deposition in extracellular matrix, most likely through a transforming growth factor-*β* pathway [16, 25]. In preclinical setting, the infusion of galectin-3 in the pericardial sac of normal animals was associated with the development of significant myocardial fibrosis, cardiac remodeling, and subsequent heart failure. The results from the same research group further suggested that levels of galectin-3 correlated significantly with the degree of myocardial fibrosis [26]. Moreover, a significant reduction in cardiac fibrosis occurred with genetic or pharmacological inhibition of galectin-3, further substantiating a central role of galectin-3 in promoting profibrotic processes in the failing myocardium [27, 28]. In the clinical setting, galectin-3 has been established as a biomarker of adverse left ventricular remodeling and heart failure morbidity and mortality [13–15]. Our data further showed that galectin-3 plasma levels in patients with ischemic and nonischemic heart failure do not differ significantly. This is in line with our previous observations that showed no differences in the myocardial scar burden (assessed by electromechanical mapping) in the two patient cohorts (53 ± 18% in ICMP vs. 55 ± 23% in DCMP; *p* = 0.83), suggesting a common pathophysiological pathway despite different initial injury to the myocardium [10]. Although direct histochemical correlation of scar burden and galectin-3 in humans is lacking, galectin-3 has been shown to correlate with the presence of myocardial replacement fibrosis as assessed by cardiac MRI using late gadolinium enhancement approach [29]. Despite this hypothesis, we failed to demonstrate a correlation between baseline galectin-3 levels and response to CD34+ cell therapy. This is in line with the observation that although levels of galectin measured in myocardial tissue samples correlate well with the degree of myocardial fibrosis the correlation of circulating galectin-3 levels and cardiac fibrosis is not as strong [30].

Cardiac fibrosis is a basic constituent of most cardiac pathologies and represents a universal response of the myocardium to all forms of cardiac injury [31]. Although essential to the healing process, enhanced extracellular matrix synthesis and collagen I deposition have been associated with increased myocardial stiffness, interruption of cardiomyocyte electrical coupling, and diminished oxygen and nutrient flow, aggravating the remodeling of the failing myocardium [32, 33]. Interestingly, despite significant impact of cardiac fibrosis on cardiac structure, function, and patient prognosis, current medical and device-based treatment options have largely failed to inhibit cardiac fibrosis formation and replace the lost cardiomyocyte mass with the new structurally and functionally integrated contractile cells.

Recently, several preclinical and clinical studies have consistently demonstrated that stem cell therapy improves myocardial perfusion and reduces scar burden in the failing myocardium [6, 7, 34, 35]. Caduceus trial showed that 12 months after intracoronary infusion of cardiosphere-derived cells (Stem Cell Group), myocardial scar burden decreased by 12.3% whereas in the Controls only 2.2% scar reduction was observed (*p* = 0.001). Additionally, regional contractility improved significantly more in the Stem Cell Group than in the Controls (*p* = 0.001) [7]. These data were further corroborated by data from Prometheus trial using transepicardial injections of autologous mesenchymal stem cells. The study showed significant reduction in scar mass (−48 ± 8%, *p* < 0.00001) and improved global LVEF (+9 ± 2%, *p* = 0.0002) compared to baseline values, with improved perfusion and contractile properties of the injected segments [36]. These results are in line with our data where responders to CD34+ cell therapy showed a significant decrease in galectin-3 plasma levels and concomitant improvement in LVEF. A decrease in galectin-3 plasma levels thus very likely reflects the reduction of the myocardial scar burden in this patient subgroup. On the other hand, galectin-3 plasma levels increased in nonresponders potentially suggesting that whether or not the patient will respond to stem cell therapy may partially depend on the capacity of the injected stem cells to effectively reduce scar burden in the failing myocardium. However, given the heterogeneous patient population and different stem cell types used in these trials, this speculation should be viewed as hypothesis generating and needs to be validated in prospective settings.

Interestingly, despite nonresponders generally appearing sicker and having lower LVEF, higher serum NT-proBNP, and worse renal function, baseline galectin-3 plasma levels were not significantly different from the levels seen in responders. The most likely explanation for this apparent mismatch is that LVEF, NT-proBNP, and renal function are markers of the patient's hemodynamic and volume status whereas galectin-3 reflects structural changes in the failing myocardium which only inconsistently correlate with patient's hemodynamics [16].

This study has several limitations. For one, we have performed a post hoc analysis of the patients included in two randomized clinical trials. However, all patients received the same CD34+ cell type and were treated using the same cell mobilization protocols and transendocardial cell injection procedure using NOGA system; thus, we believe that patient population included in our study was sufficiently homogeneous for the analysis. Additionally, only 13% of our patients had galectin-3 levels higher than 17.9 ng/mL which is currently accepted as the cut-off value for worse patient prognosis which means that prognostic significance of baseline galectin-3 plasma levels on patient response to CD34+ cell therapy may have been underestimated. Also, our analysis was performed on fairly small number of patients. Finally, due to the retrospective design of the study, we were not able to perform any noninvasive imaging to quantify the degree of fibrosis in our patient cohort.

## 5. Conclusions

To date, this is the first study to explore an association between changes in galectin-3 plasma levels and response to stem cell therapy in patients with chronic heart failure. Although in the analyzed patient cohort baseline galectin-3 plasma levels did not predict the response to stem cell therapy, our data suggests that a decrease in galectin-3 plasma levels at 3-month follow-up correlates with a response to CD34+ cell therapy. This suggests that the effects of stem cells on scar burden may play a significant role in patients' response to stem cell therapy. Further prospective trials are warranted to confirm our initial findings and to deepen our understanding of galectin-3 in the field of stem cell therapy.

## Figures and Tables

**Table 1:** Baseline patient characteristics. Values are presented as mean ± SD, number of patients (percent), or median (IQR) for NT-proBNP.

	All (*N* = 61)	ICMP (*N* = 19)	DCMP (*N* = 42)	*p*
Age (y)	52 ± 12	55 ± 8	51 ± 10	0.16
Male gender (%)	51 (83)	15 (79)	36 (85)	0.54
LVEF (%)	30 ± 6	29 ± 6	30 ± 5	0.41
6MWT (m)	482 ± 82	475 ± 82	486 ± 83	0.92
NT-proBNP (pg/mL (IQR))	1132 (350-2279)	1133 (394-2239)	1127 (333-2359)	0.99
Creatinine (mmol/L)	86 ± 23	89 ± 21	84 ± 24	0.52
Bilirubin (*μ*mol/L)	16 ± 8	16 ± 6	17 ± 9	0.75
Sodium (mmol/L)	140 ± 2	140 ± 3	141 ± 2	0.63
AST (*μ*mol/L)	0.5 ± 0.1	0.45 ± 0.14	0.47 ± 0.14	0.62
AF (*μ*mol/L)	1.2 ± 0.4	1.21 ± 0.33	1.13 ± 0.39	0.39
gGT (*μ*mol/L)	1.1 ± 1.4	0.68 ± 0.38	1.2 ± 1.4	0.10
RDW (%)	13.7 ± 1.2	13.7 ± 1.1	13.7 ± 1.2	0.92
Leukocytes (×109)	7.1 ± 1.6	6.9 ± 1.4	7.2 ± 1.6	0.47
Hemoglobin (g/L)	144 ± 11	146 ± 10	143 ± 12	0.32
Platelet count (×109)	208 ± 49	198 ± 34	213 ± 53	0.27
Medical management				
ACEI/ARB	61 (100)	19 (100)	42 (100)	/
Beta blockers	61 (100)	19 (100)	42 (100)	/
MRA	61 (100)	19 (100)	42 (100)	/
Diuretic	33 (54)	12 (63)	21 (51)	0.34
Aspirin	21 (34)	19 (100)	2 (4)	<0.05
Galectin-3 levels (ng/mL)				
Baseline	13.4 ± 5.5	15.1 ± 7.2	12.7 ± 4.3	0.12
3 months	13.1 ± 5.8	13.3 ± 5.8	12.4 ± 5.8	0.83

ICMP: ischemic cardiomyopathy; DCMP: nonischemic cardiomyopathy; LVEF: left ventricular ejection fraction; 6MWT: 6-minute walk test; AST: aspartate transaminase; AF: alkaline phosphatase; gGT: *γ*-glutamyltranspeptidase; RDW: red cell distribution width; ACEI: angiotensin convertase inhibitor; ARB: angiotensin receptor blocker; MRA: mineralocorticoid receptor antagonist.

**Table 2:** Comparison of characteristics of responders and nonresponders to CD34+ cell therapy. Values are presented as mean ± SD, number of patients (percent), or median (IQR) for NT-proBNP.

	Nonresponders (*N* = 18)	Responders (*N* = 43)	*p*
Age (y)	55 ± 10	51 ± 13	0.32
Male gender (%)	15 (85)	36 (84)	0.96
Nonischemic CMP (%)	13 (72)	29 (67)	0.67
LVEF (%)			
Baseline	27 ± 6	31 ± 5	<0.05
3 months	28 ± 6	39 ± 7	<0.05
6MWT (m)	464 ± 101	490 ± 73	0.28
NT-proBNP (pg/mL (IQR))	3245 (1584-4336)	609 (240-1323)	<0.05
Creatinine (mmol/L)	101 ± 30	79 ± 16	<0.05
Bilirubin (*μ*mol/L)	17 ± 9	16 ± 8	0.48
Sodium (mmol/L)	140 ± 2	141 ± 2	0.84
AST (*μ*mol/L)	0.5 ± 0.1	0.4 ± 0.1	0.10
AF (*μ*mol/L)	1.2 ± 0.4	1.1 ± 0.4	0.60
gGT (*μ*mol/L)	1.2 ± 1.3	1.1 ± 1.4	0.68
RDW (%)	14.3 ± 1.4	13.4 ± 0.9	0.01
Leukocytes (×109)	7.1 ± 1.9	7.1 ± 1.4	0.85
Hemoglobin (g/L)	142 ± 14	144 ± 10	0.44
Platelet count (×109)	207 ± 69	209 ± 37	0.89
Medical management			
ACEI/ARB	18 (100)	42 (100)	/
Beta blockers	18 (100)	42 (100)	/
MRA	18 (100)	42 (100)	/
Diuretic	12 (67)	21 (48)	0.24
Aspirin	7 (39)	26 (60)	0.13
Galectin-3 levels (ng/mL)			
Baseline	13.2 ± 4.9	13.6 ± 5.7	0.80
3 months	15.7 ± 8.4	12.1 ± 4.0	<0.05

ICMP: ischemic cardiomyopathy; DCMP: nonischemic cardiomyopathy; LVEF: left ventricular ejection fraction; 6MWT: 6-minute walk test; AST: aspartate transaminase; AF: alkaline phosphatase; gGT: *γ*-glutamyltranspeptidase; RDW: red cell distribution width; ACEI: angiotensin convertase inhibitor; ARB: angiotensin receptor blocker; MRA: mineralocorticoid receptor antagonist.

**Table 3:** Multivariate predictors of response to CD34+ cell therapy.

Variable	*B* coefficient	*p* value	95% confidence interval	
			Lower bound	Upper bound
LVEF < 30%	0.122	0.220	-0.075	0.319
CKD	-0.201	0.120	-0.456	0.054
NT‐proBNP > 1000 pg/mL	-0.245	0.031	-0.467	-0.023
RDW > 14.5%	-0.121	0.330	-0.367	0.126
Galectin-3 decrease	0.306	0.003	0.113	0.498

LVEF: left ventricular ejection fraction; CKD: chronic kidney disease; RDW: red cell distribution width.

## Data Availability

The data used to support the findings of this study are available from the corresponding author upon request.
